# Autoimmune Hepatitis: Single-center Experience of Clinical Presentation, Response to Treatment and Prognosis in Saudi Arabia

**DOI:** 10.4103/1319-3767.61235

**Published:** 2010-04

**Authors:** Hind I. Fallatah, Hisham O. Akbar, Yousif A. Qari

**Affiliations:** Gastroenterology Unit, Department of Medicine, King Abdul Aziz University Hospital, Jeddah, Saudi Arabia

**Keywords:** Autoimmune hepatitis, clinical presentation, laboratory features, Saudi Arabia, treatment

## Abstract

**Background /Aim:**

Autoimmune hepatitis (AIH) is a common cause of end-stage liver disease worldwide. It is a disease prevalent in children and adults, with female predominance and variable clinical presentations. AIH has favorable responses to steroids and immunomodulators. Diagnosis of AIH is based on clinical and laboratory criteria, as suggested by the International Autoimmune Hepatitis Group. Data on the disease pattern of AIH from the Middle East countries is scarce.

**Materials and Methods:**

In this retrospective analysis, we studied clinical and laboratory features, immunological data, radiological findings, liver biopsy findings and response to therapy in patients with AIH from the hepatology clinics of King Abdul Aziz, University Hospital, Jeddah, from 1994 to 2008.

**Results:**

We diagnosed 41 patients with AIH, and 33 were included in the analysis. The mean age was 32.3 years, with female predominance of 75.7%. De-compensated cirrhosis at presentation was found in 45.5% of the patients. Acute hepatitis was associated with significantly higher levels of the serum ALT and bilirubin (*P*=0.001 and *P*=0.03, respectively). All our patients had type 1 AIH. Treatment with prednisolone and azathioprine resulted in complete or partial remission in majority of the patients (54.8%). However, patients with advanced disease showed a poorer response to treatment (*P*=0.016). Six patients with poor compliance had relapse of AIH. Two patients had a flare of the disease during pregnancy, and they responded well with prednisolone. The longest follow-up was 14 years and the shortest was 2 months. Four patients died from liver disease.

**Conclusion:**

AIH patients in Saudi Arabia are likely to present with advanced disease at a young age and would have a poorer response to therapy as compared with patients in other countries worldwide.

Autoimmune hepatitis (AIH) is one of the leading causes of liver cirrhosis and end-stage liver disease worldwide. The international prevalence of AIH among patients with liver disease is between 11% and 20%.[1] It is usually a disease of children and young-to-middle age females,[2,3] but it can also affect individuals with older age and males.[4,5] The clinical presentations of the disease are variable, from asymptomatic abnormal liver enzymes to fulminant liver failure or advanced decompensated cirrhosis.[2,5,6] Diagnostic scoring system for AIH has been established since 1993 by the International Autoimmune Hepatitis Group[7]; it was revised and updated in 1999,[8] and most recently a simplified criteria were established in 2008.[9]

Treatment of AIH with steroids and immunomodulators will result in remission in most patients.[2,4,10] The prognosis of AIH is variable according to the disease severity and progression, although generally the disease has favorable outcome, with 10 years survival to liver-related death or liver transplant of 83.3% and 89.5% in asymptomatic and symptomatic patients, respectively, and 23 years cumulative transplant-free survival of 73.5%.[2,11,12] Post liver transplant, AIH patients have a 5-year survival rate, similar to that in patients with genetic liver disease.[13] In Asia and the Middle East countries, including Saudi Arabia, AIH may be under-diagnosed or overlooked by the high prevalence of chronic hepatitis B and C.[14,15] Fewer reports have been published about AIH in Asia as compared to Europe and North America.[6] Apart from a few reported cases, there are no published local data about the clinical presentations and prognosis of AIH in Saudi Arabia. In the following retrospective cohort study, we reported clinical presentations, laboratory results, responses to therapy and prognostic outcomes of patients with AIH admitted to the King Abdul Aziz University Hospital (KAUH), which is the main university hospital in Jeddah, Saudi Arabia.

The aim of the study was to investigate the clinical patterns and laboratory and immunological features of AIH, and also to assess the responses to therapy and prognosis of AIH patients at KAUH in Jeddah, Saudi Arabia.

## MATERIALS AND METHODS

We performed a retrospective analysis of all patients diagnosed to have AIH, based on the criteria of the International Group of Autoimmune Hepatitis,[7,8] from the hepatology clinics of KAUH from 1994 to 2008. For each patient, age, sex, clinical presentation at diagnosis, laboratory and immunological data were obtained. Liver biopsy results were also included if available. The data of the responses to therapy and the outcome at the end of the follow-up period were also collected. Patients were excluded from the analysis if evidence with regard to a diagnosis of AIH was insufficient; if the medical record was not complete because of poor follow-up; if the patients had other coexisting liver disease, e.g., nonalcoholic fatty liver disease (NFLD) or chronic hepatitis C (CHC). Clinical presentations were categorized as asymptomatic, defined as abnormal liver enzymes for more than 6 months with positive immunological data; liver biopsy features suggestive of AIH; and absence of other causes of liver disease, including drugs. Acute hepatitis was defined as acute symptoms, including fever, jaundice and right upper abdominal pain, with serum alanine aminotransferase more than 500 U/L. Decompensated cirrhosis was defined as the presence of one of the following features: ascites, variceal bleeding, hepatic encephalopathy, bacterial peritonitis, low serum albumin and prolonged prothrombin time (PT). Presence of other coexisting autoimmune diseases (AIDs) was also researched.

Laboratory data included the following: Liver functions tests were assessed by the dimension clinical chemistry system Flex reagent cartridge (serum alanine aminotransferase [ALT; normal, 30–65 U/L], aspartate amino transferase [AST; normal, 15–37 U/L], alkaline phosphatase [ALP; normal, 50–136 U/L], gamma-glutamyl transferase [GGT; normal, 5–85 U/L], total protein [TP; normal, 64–82 g/L], albumin [Alb; normal, 35–50 g/L], total and direct bilirubin [normal, 0–17 and 0–5 µmol/L, respectively]). A complete blood count (CBC) was done at the time of diagnosis: (white blood cells [WBC; normal, 3–11 KU/L], hemoglobin [Hg; normal, 12–17 g/dL] platelet count [plate; normal, 100–400 KU/L]). Cytopenia caused by hypersplenism was considered to be present if the WBC count was less than 3 (109/*µ*L) and/ or platelet count was less than 100 (103/*µ*L) in the in the presence of splenomegaly on abdominal ultrasound or CT examination.

Hepatitis serology was performed by ELISA (enzyme-linked immunosorbent assay) for hepatitis B virus (HBSAg, HBeAg, HBeAb, HBcAb) and hepatitis C virus (HCVAb) in all patients; also in patients with acute presentation, the results of hepatitis A virus (HAVAb-IgM) and hepatitis E virus (HEVAb if available) were obtained.

Results of testing for Wilson's disease by 24-hour urine copper and serum copper were determined, and results of transferrin saturation for possible hemochromatosis were reviewed. We also obtained the result of immunological assessment for antinuclear antibody (ANA) performed by indirect immunofluorescence (IIF)—weakly positive- 1/40 and strongly positive- 1/1280; smooth muscle antibody (SMA) was detected by ELISA; liver kidney microsomal-1 (LKM-1) was detected by ELISA; antimitochondrial (AMA) was detected by ELISA; immunoglobulin-G (IgG) level was determined by the nephelometer method (normal range, 5.4–16.1); and anti-neutrophil cytoplasmic antibody (ANCA) was detected by IIF.

The results of upper abdominal ultrasound and/ or abdominal computed tomography (CT) scan at the time of diagnosis were also reviewed. Upper gastrointestinal endoscopic examination result at presentation was obtained (presence of esophageal or gastric varices and/ or portal hypertensive gastropathy was considered as decompensated portal hypertension) even in the absence of a history of bleeding. For patients who had cholestasis at presentation, the results of endoscopic retrograde cholangiopancreatography (ERCP) were included. Liver biopsy results if available were also obtained.

### Data regarding treatment and follow-up

Data was collected on modes of treatment, starting and maintenance doses, duration of treatment, treatment response, treatment withdrawal, side effect of medication; and information about medication if any apart from prednisolone and azathioprine (AZA) added to control the disease was obtained.

ALT and serum bilirubin results at diagnosis and at 3, 6, 12 and 24 months after starting treatment (if available) and at the end of the follow-up period were obtained.

Response to treatment was considered complete if the serum ALT level dropped to normal range within 6-24 months of treatment, together with normalization of the serum bilirubin if it was elevated before the treatment. Reduction of ALT to a level below the normal range after 24 months of treatment or by the end of follow-up period if the follow-up period was less than 2 years was considered as incomplete response. Patients that failed to achieve reduction of the serum ALT level and bilirubin or had an elevation in these within the 24 months were considered as nonresponders.

Relapse was defined as elevation of ALT to above normal or to pre-treatment level after an initial response. Duration of follow-up for each patient was recorded. Patients who had progression to decompensated cirrhosis during the follow-up period were identified. Mortality was defined as death during the follow-up period.

### Statistical method

Statistical package for social sciences (SPSS) for Windows version 15 was used. The means, standard deviations and frequencies were defined. The chi-square test was used to assess the relation between categorical variables. The independent sample *t* test was used to correlate the serum ALT and bilirubin to the severity of liver disease. *P* value of less than 0.05 was considered significant.

## RESULTS

**Figure 1:**
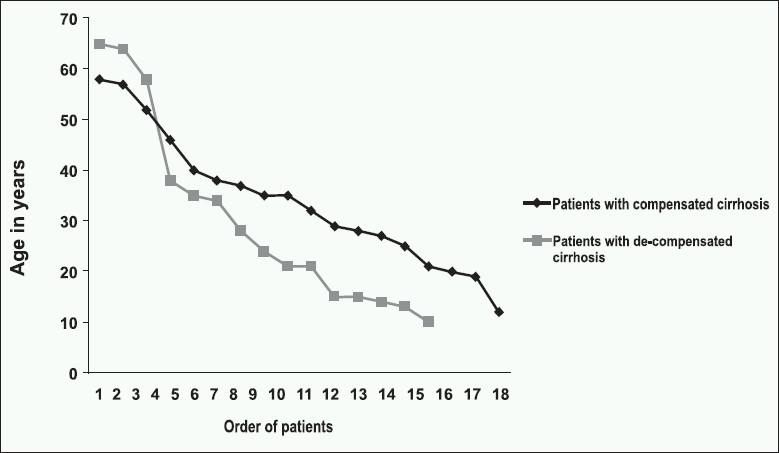
Age in relation to de-compensated cirrhosis

From July 1994 to June 2008, 41 patients were diagnosed to have AIH; 33 patients were included in the analysis. Eight patients were excluded. The mean age at presentation was 32.3 years (range, 10–65 years), and 22 (66.66%) patients were aged 35 years or younger. Twenty-five (75.7%) patients were female and 8 were male. Sixty percent of the patients were Saudi. Twelve (36.4%) patients had acute hepatitis at presentation. Jaundice was the most common symptom; however, 7 patients were asymptomatic. Table 1 lists symptoms at presentation. Fifteen (45.5%) patients had decompensated cirrhosis [Figure 1]. Six patients had hypersplenism (thrombocytopenia or leukopenia or both). Liver functions varied from modest elevation in ALT and serum bilirubin to very high levels (*P*=0.000 and .03, respectively), in patients with acute hepatitis in comparison to those with asymptomatic and chronic patients. Three patients had cholestasis (higher ALP and GGT compared to ALT and AST) at presentation. Twenty-six patients had serum IgG level result available at the time of diagnosis—1 had normal level, 9 had mild elevation (less than 1.5 times the normal) and 16 had levels more than 1.5 times the normal. Results of the baseline laboratory results are shown in Table 2. ANA result at diagnosis was available for 31 patients; it was mildly positive in 12 patients, moderate in 11 and strongly positive in 8 patients. Smooth muscle antibody (SMA) result was available for 31 patients; it was negative in 7 patients, moderately positive in 17, and strongly positive in 7 patients. AMA was negative in all the patients. Result of LKM-1 was available for 27 patients; it was negative in all of them.

**Table 1:** Symptoms at presentation

**Symptom**	**Number and percent of patients**
Asymptomatic	7 (21.2)
Jaundice	17 (51.5)
Ascites	7 (21.2)
Encephalopathy	4 (12.1)
Fatigue	2 (6.06)
Bacterial peritonitis	2 (6.06)

**Table 2:** Baseline laboratory results

	**Median**	**Range**
ALT IU/L	206	25–1542
AST U/L	414	29–1507
Serum bilirubin µmol/L	106.5	3–584
Alkaline phosphatase U/L	206	110–684
GGT U/L	156	24–1256
Serum albumin g/L	29.5	12–41
INR (seconds)	1.45	0.9–3.4
IGG level	28.8	14.3–61.3
Platelets count K/µL	221	28–453

ALT: Alanine aminotransferase; AST: Aspartate amino transferase; GGT: Gamma-glutamyl transferase; IGG: Immunoglobulin-G; INR: international normalized ratio

Hepatitis serology was negative in all patients for HBV and HCV and HAV (in patients with acute presentation); only 3 patients were tested for HEVAb, and it was negative. One third of the patients were tested for PANCA, and it was positive only in 2 patients. Abdominal ultrasound and/or CT scan results on presentation were available for 31 patients; 17 had normal examination, 6 had evidence of cirrhosis without ascites and 8 had advanced cirrhosis with ascites. Only 12 patients had liver biopsy performed at the time of diagnosis, which was consistent with AIH in all of them. In 15 patients with decompensated cirrhosis at presentation, liver biopsy was not performed. Three patients had liver biopsy performed at other centers, for which no results were available in our records; and 2 patients refused liver biopsy. Upper gastrointestinal endoscopy at diagnosis was performed in 29 patients: 21 were normal, 6 had esophageal varices and 2 had portal hypertensive gastropathy. Six patients had coexistent other autoimmune disease/diseases: 3 had systemic lupus erythematosus (SLE), 2 had rheumatoid arthritis and 1 had SLE and Hashimoto's disease.

### Treatment

Thirty-one patients received treatment. Two patients were not treated due to persistently normal liver enzymes: one had only transient elevation of ALT (to 100 U/L) at the time diagnosis of AIH, and then dropped spontaneously to normal; and the other had decompensated cirrhosis at presentation. The starting dose of prednisolone was 25–40 mg/day, and maintenance dose was 5–15 mg/day. AZA was added when the response to prednisolone was incomplete or as a steroid-sparing agent after response to prednisolone. Starting dose of AZA was 50–100 mg/day, and maintenance dose was 50–100 mg/day. Seventeen (54.8%) of the treated patients (13 compensated and 4 decompensated cirrhosis) had complete response, 11 patients (35.48% [7 of them had decompensated cirrhosis]) had incomplete response and 3 decompensated cirrhosis patients had no response to the treatment. Duration to complete response varied from 1 to 20 months, with a mean of 5 to 6 months. In general, patients with advanced cirrhosis were less likely to respond to treatment (*P* value, 0.016). Two patients with advanced cirrhosis did not respond and the treatment was withdrawn by the physician. One patient with complete response developed a disease flare after 5 years of maintenance therapy when treatment was interrupted, and was subsequently resumed on treatment. Nine patients stopped treatment on several occasions; 4 of them had one relapse and 2 had frequent relapses. Medication in 1 patient who had AZA-induced pancytopenia was shifted to mycophenolate mofetil (MMF) 750 mg twice daily after the second relapse. Four patients had a relapse while on maintenance treatment, and 3 of them had decompensated cirrhosis. Seventeen patients had side effects from prednisolone or AZA or both [Table 3]. As expected, diabetes was the most common side effect. Two patients progressed to decompensated cirrhosis during the follow-up period; both of them had variceal bleeding, hypersplenism and encephalopathy, and both were referred for liver transplant. Four patients died from liver-disease–related complications, and 1 died during pregnancy as a result of pregnancy-related complication. Though not statistically significant, patients with advanced cirrhosis had higher chance of death during the follow-up period. The longest duration of follow-up was 14 years and the shortest was 2 months (1 patient died with fulminant acute AIH); while 69.7% of these patients were on follow-up for more than 2 years, with a mean of 4 years.

**Table 3:** Treatment side effects

**Treatment side effects**	**Frequency (%)**
None	18 (54.5)
Diabetes	4 (12.1)
Proximal myopathy	5 (12.1)
Pancytopenia	1 (3.0)
Infections	4 (12.1)
Total	31 (93.9)
No treatment	2 (6.1)
Total	33 (100)

Twenty-four of the 28 survivors are still on active follow-up at our hepatology clinics.

## DISCUSSION

The prevalence of AIH among patients with liver disease in Saudi Arabia is not known;[16] it may be much less as compared to North America and European countries. Kalaf and colleagues in their review of 112 liver transplantations (LTs) in Riyadh found that 14.3% of LT indications were due to AIH.[17] In our cohort, 22 patients were aged less than 35 years; 50% of them had decompensated cirrhosis at presentation. This is in contrast to previously reported data from Japan and the United States in which older patients were reported to have more advanced disease.[4,5] It is even higher than the rate reported from an Indian study (34.2%).[6] Acute hepatitis at presentation was observed in 36.4% of our patients, either newly diagnosed or a flare of established cirrhosis. This is comparable to the North American and European figures of 26% to 40%;[18,19] but on the other hand, significantly higher than the Indian rate of 13.1%.[6] Asymptomatic patients were found to be more likely to have lower serum ALT compared to symptomatic patients (*P* value, 0.05). This is similar to the data reported by Feld and colleagues.[12] Three females had cholestasis at presentation, and they were thought to have primary biliary cirrhosis although their autoimmune profiles were consistent with AIH; and 2 of these patients had liver biopsy features of AIH. Those three female patients had good initial response to treatment and long-term follow-up compared to patients with hepatocellular pattern at presentation. Huang and colleagues from Taiwan reported similar data on AIH patients with cholestasis.[20] Present research reported positive ANA in 87.8% of patients and positive SMA in 72.7%. This is higher than the figure reported by Czaja, 67% for ANA and SMA together.[21] In the most recently published criteria for diagnosis of AIH by Hennes and colleagues, it was found that serum IgG of 1.44, the upper normal limit (UNL), is the best diagnostic predictor for AIH.[9] In our cohort, serum IgG level was more than 1.44 (UNL) in 61.5% of the patients. All our patients had type 1 AIH; none of them had positive LKM-1; but in previous reports, 4% to 20% of patients had LKM-1–positive type II AIH.[19] Older patients with AIH were more likely to have coexisting autoimmune diseases, similar to what has been reported by Czaja and Carpenter reported similar findings.[5] One patient had disease onset during pregnancy, and 2 other patients had flare of the disease during pregnancy; all of them had remission on prednisolone and favorable outcome of pregnancy. Similar outcome of AIH with an onset during pregnancy was reported by Floreani and his colleagues.[2] We had 54.8% complete response rate, which is lower than internationally reported complete response rate of 65% at 18 months and 80% at 3 years.[19] Neither the initial ALT level nor the duration of symptoms predicted the response, but decompensated patients were found to be less likely to respond as compared to compensated patients (*P* value, 0.016). Fulminant forms of AIH have high mortality in untransplanted patients;[22] in our cohort, one 64-year-old woman had fulminant AIH. Data on MMF in AZA-intolerant AIH patients showed 43% remission rate;[23] 1 patient who had AZA-induced pancytopenia responded well to MMF. Genetic factors are thought to influence the disease severity in AIH.[24] This may be the reason behind different clinical patterns and severity levels of AIH in our patients as compared to patients from other countries.

Our study is limited by the relatively small number of patients; multicenter national data are needed. To overcome the other limitation that data were retrospectively collected, a prospective, well-structured study with complete data of patients would give more accurate local figures of AIH.

## CONCLUSION

These results showed that many of our AIH patients are young, with advanced disease at presentation and poor response to treatment, as compared to patients in other countries. Early recognition and treatment of AIH in Saudi Arabia is essential to avoid complications of liver cirrhosis and to reduce the need for liver transplant, in medically treatable disease conditions. National data about AIH from different regions of the country are needed for better understanding of the local disease prevalence among patients with chronic liver disease; for identification of clinical and laboratory patterns; and for assessing the response to therapy.
